# Impact of a physical–cognitive intervention on emotional regulation and school adjustment in primary school children

**DOI:** 10.3389/fpsyg.2026.1790797

**Published:** 2026-08-13

**Authors:** María del Carmen Carcelén-Fraile, María Luisa Montánchez-Torres

**Affiliations:** 1 Department of Educational Sciences, Faculty of Social Sciences, University of Atlántico Medio, Las Palmas de Gran Canaria, Spain; 2 International Scientific Association on Innovation in Education and Health (ACIINES), Jaén, Spain; 3 International Network of Educational Law, Las Palmas de Gran Canaria, Spain; 4 Department of Educational Sciences, Faculty of Education, International University of La Rioja, Logroño, Spain

**Keywords:** emotional regulation, motor games, primary education, school adjustment, self-regulation, soroban abacus

## Abstract

**Introduction:**

Self-regulation, emotional regulation, and school adjustment are fundamental for children’s learning and wellbeing during primary education. This study aimed to analyze the effects of a combined physical–cognitive intervention based on motor games and soroban abacus training on these outcomes in children in the first cycle of primary education.

**Methods:**

A randomized controlled trial was conducted with 82 primary school children allocated to an experimental group (*n* = 41) or a control group (*n* = 41). The experimental group completed a 12-week physical–cognitive intervention integrated into physical education classes, whereas the control group followed the standard curriculum. Self-regulation, emotional regulation, and school adjustment were assessed before and after the intervention using validated questionnaires.

**Results:**

Compared with the control group, the experimental group showed significant improvements in self-regulation, emotional regulation, and all dimensions of school adjustment, including emotional symptoms, behavioral problems, peer relationship problems, hyperactivity/inattention, and prosocial behavior. Moderate to large effect sizes were observed across the primary outcomes.

**Discussion:**

These findings suggest that integrating motor games with soroban-based cognitive training may represent an effective educational strategy for enhancing self-regulation, emotional regulation, and school adjustment in young children. Although the results are promising, they should be interpreted cautiously due to the relatively small sample size and the exploratory nature of some secondary analyses.

## Introduction

In recent decades, the study of cognitive and emotional processes in primary school children has gained increasing interest due to their significant influence on academic performance, social adjustment, and overall wellbeing (Zhao and Sang, 2025; Schonert-Reichl et al., 2015). Children’s ability to self-regulate their emotions and behaviors plays a crucial role in their development and in how they cope with academic and social challenges (Adynski et al., 2024). Self-regulation and emotional regulation are considered fundamental processes for effective learning, as they allow children to manage stress, maintain attention, and persevere in long-term tasks, all of which are essential for their school adjustment and academic success (Eisenberg et al., 2010; Simón-Grábalos et al., 2025).

Self-regulation refers to the ability of individuals to manage their thoughts, emotions, and behaviors in relation to long-term goals (Nakhostin-Khayyat et al., 2024). In the school context, self-regulation translates into the ability to maintain concentration, control impulses, and adapt to the academic and social demands of the educational environment (Akeren et al., 2025). Emotional regulation, on the other hand, is the ability to identify, understand, and manage emotions appropriately in various situations, which is closely related to the quality of social interaction and school adjustment (Lopes et al., 2011). The importance of these skills in the school context is crucial, as children who struggle to regulate their emotions or behavior often face both academic and social challenges (Paulus et al., 2021), which can lead to poor school adjustment and, in more severe cases, school dropout (Hosokawa et al., 2024). Therefore, the implementation of effective interventions that foster the development of these key skills is essential.

Among the emerging strategies for promoting self-regulation and emotional regulation in children, the combination of physical and cognitive activities has been proposed (Bai et al., 2021). Physical activity not only improves physical health but also has beneficial effects on the brain, promoting memory, attention, and emotional control (Mandolesi et al., 2018). In particular, motor games have proven effective in improving motor control, concentration, and social skills, creating an environment conducive to learning and emotional management (Fizi et al., 2023). Motor games, which include structured physical activities such as team games or coordination exercises, not only promote physical wellbeing but also foster self-regulation and emotional adaptation, both of which are necessary for academic success (Wang and Zhou, 2024).

On the other hand, cognitive training, particularly through the use of the soroban abacus, has proven to be a powerful tool for improving children’s attention, concentration, and problem-solving skills (Takaoka et al., 2024; Li et al., 2016). The soroban, an abacus of Japanese origin, promotes cognitive development by requiring children to perform complex mental calculations, thereby improving their ability to plan, monitor, and regulate their cognitive processes efficiently (Lima-Silva et al., 2021). Using the soroban not only trains mathematical skills but also fosters cognitive self-regulation by requiring children to manage their attention and maintain concentration during activities (Wang, 2020; Xu et al., 2025).

Previous research has demonstrated that physical activity interventions and cognitive training programs may independently contribute to improvements in executive functioning, emotional competence, and academic performance in children (Diamond and Ling, 2016; Wang and Zhou, 2024). Likewise, several combined physical–cognitive interventions have shown positive effects on attention, working memory, inhibitory control, and self-regulation (Rondão et al., 2022; Ouyang et al., 2025). In parallel, studies examining abacus-based training have reported improvements in executive functioning, response inhibition, and mathematical processing abilities in school-aged children (Na et al., 2015; Wang, 2020; Takaoka et al., 2024). However, most previous interventions have primarily focused on neurocognitive or academic outcomes and have rarely examined the combined effects of integrated motor–cognitive programs on emotional regulation and school adjustment during early primary education. Furthermore, limited evidence exists regarding interventions specifically combining cooperative motor games with soroban-based mental calculation training within the same instructional sequence. Therefore, the present study aimed to address this gap by examining the effects of an integrated physical–cognitive intervention on self-regulation, emotional regulation, and school adjustment in primary school children. This study aimed to examine how a combined intervention based on motor games and cognitive training using the soroban abacus affects three key domains: self-regulation, emotional regulation, and school adjustment. Through a controlled experimental design, the effects of this intervention on children will be evaluated before and after implementation, providing empirical evidence on the effectiveness of this approach in improving emotional wellbeing and adaptation to the school environment.

## Methods

### Participants

**Figure 1:**
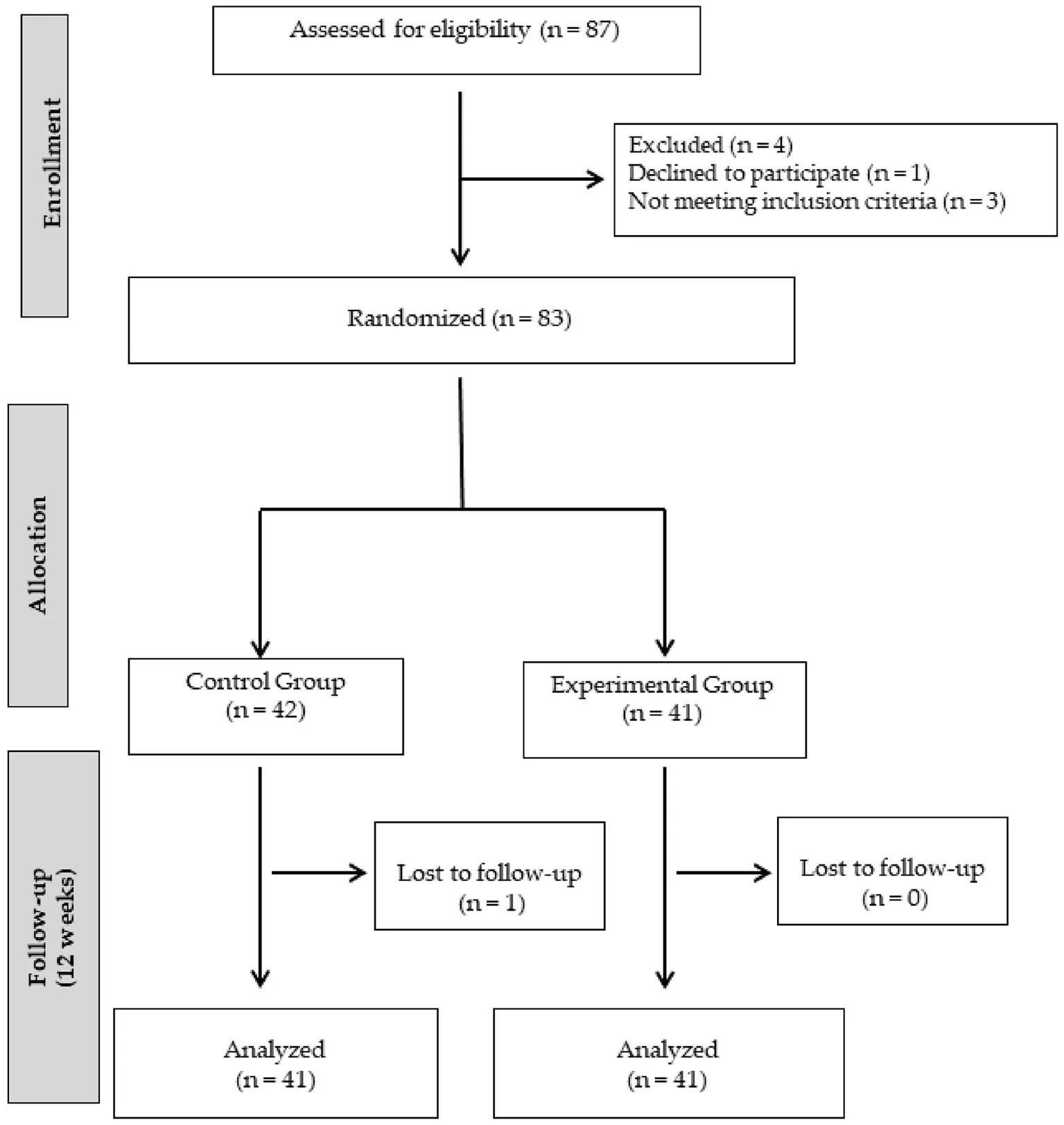
Participant flowchart.

For this study, 90 children in the first cycle of primary education were invited to participate. Of these, 83 met the predefined eligibility criteria and provided written informed consent signed by their parents or legal guardians. The inclusion criteria were as follows: (i) school-aged children enrolled in the first cycle of primary education; (ii) children capable of engaging in moderate physical activity without medical restrictions and able to use the soroban abacus; and (iii) voluntary participation with prior informed consent from parents or guardians. Exclusion criteria included: (i) health conditions preventing safe participation in physical activities or limiting the use of the abacus (e.g., heart disease, severe respiratory disorders, or motor disabilities); (ii) severe cognitive or learning disorders that could significantly interfere with the intervention; and (iii) participation in extracurricular programs involving similar physical or cognitive training. After baseline assessment, participants were randomly assigned to the experimental group (*n* = 41) or the control group (*n* = 42). During the post-intervention assessment, two participants from the control group did not complete the evaluation procedures and were excluded from the final analyses. Therefore, the final analyzed sample consisted of 82 children (41 in the experimental group and 41 in the control group), with a mean age of 6.73 ± 0.95 years, of whom 48 were boys (58.5%) and 34 were girls (41.5%) (Figure 1; Table 1).

**Table 1:** Description of the sociodemographic and clinical characteristics of the participants.

Variables		Total (*n* = 82)	Experimental (*n* = 41)	Control (*n* = 41)	*p*-value
Age (years)		6.67 ± 1.02	6.68 ± 1.04	6.66 ± 1.01	0.848
Sex	Boy	48 (58.50)	23 (47.90)	25 (52.10)	0.394
	Girl	34 (41.50)	18 (52.90)	16 (47.90)	
Number of siblings		1.30 (0.72)	1.39 (0.70)	1.22 (0.73)	0.501
Birth order, *n* (%)	Only child	21 (25.6)	8 (9.8)	13 (15.9)	0.563
	Oldest	17 (20.7)	11 (13.4)	6 (7.3)	
	Middle	27 (32.9)	12 (14.6)	15 (18.3)	
	Youngest	17 (20.7)	10 (12.2)	7 (8.5)	
Family employment status, *n* (%)	Both parents employed	43 (52.4)	23 (28.0)	20 (24.4)	0.467
	One parents employed	39 (47.6)	18 (22.0)	21 (25.6)	

Quantitative variables are presented as mean and standard deviation. Qualitative variables are presented as frequency and percentage.

### Outcomes

Sociodemographic information was collected from participants before the intervention. This data included aspects such as gender, age, parents’ employment status, number of siblings, and birth order, in order to better understand the characteristics of the study group. Data collection was carried out by a researcher who was not involved in the design or implementation of the intervention.

#### Self-regulation

Self-regulation was assessed using the Spanish adaptation of the Self-Regulation Questionnaire for Children (SRQ-C) developed by Conesa and Duñabeitia (2022), based on the original instrument proposed by Brown et al. (1999). The questionnaire has demonstrated adequate psychometric properties in school-aged children, including satisfactory internal consistency (Cronbach’s *α* > 0.80) and evidence of construct validity for the assessment of self-regulatory processes in educational settings. The questionnaire was completed individually by the children under the supervision of a researcher trained in the assessment procedures, who provided standardized instructions when necessary. The version used in this study consists of 22 items, answered using a five-point Likert scale ranging from 1 (never) to 5 (always). The questionnaire assesses aspects related to behavioral planning, self-monitoring, task persistence, and the ability to complete initiated activities. The total self-regulation score was obtained by summing the responses to the 22 items, resulting in a score range from 22 to 110 points. Higher scores indicate greater levels of self-regulation. The total score was used as a continuous variable in the statistical analyses. The SRQ-C has demonstrated adequate psychometric properties in school-aged populations, with satisfactory internal consistency reported in previous studies. In the present study, the instrument showed a reliability of 0.736, measured using Cronbach’s alpha coefficient.

#### Emotional regulation

Emotional regulation was assessed using the Spanish validated version of the Emotion Regulation Checklist (ERC) adapted by Lucas-Molina et al. (2022) from the original scale developed by Shields and Cicchetti (1997). The instrument has shown adequate psychometric properties in child populations, including satisfactory internal consistency for both the emotional regulation and lability/negativity dimensions (Cronbach’s *α* values above 0.70), as well as appropriate convergent validity indicators. The instrument was completed by the participants’ primary classroom teachers, who remained the same raters across pre- and post-intervention assessments to ensure consistency in the evaluation process. The ERC consists of 24 items, organized into two dimensions: emotional regulation and lability/negativity. Items are answered using a four-point Likert scale, ranging from 1 (never) to 4 (almost always). In this study, both subscales of the ERC were used. The emotional regulation subscale, composed of 8 items, assesses the child’s ability to manage and appropriately modulate their emotions. A score is obtained by summing the item scores, with a range of 8 to 32 points, where higher scores indicate better emotional regulation. The lability/negativity subscale, comprised of 16 items, assesses emotional instability and the presence of intense negative emotions, with a score range of 16 to 64 points, where higher scores reflect greater levels of emotional lability. Scores from both subscales were used as continuous variables in the statistical analyses. The ERC has demonstrated adequate psychometric properties in children, with good levels of reliability and validity. In the present study, this questionnaire showed satisfactory internal consistency, as assessed by Cronbach’s alpha coefficient of 0.842.

#### School adjustment

School adjustment was assessed using the Spanish version of the Strengths and Difficulties Questionnaire (SDQ) (Goodman, 1997). Previous studies conducted in Spanish child and adolescent populations have reported adequate psychometric properties, including satisfactory internal consistency, factorial validity, and validated cut-off scores for behavioral and emotional difficulties (Rodríguez-Hernández et al., 2014; Ortuño-Sierra et al., 2022). The questionnaire was completed by the participants’ primary classroom teachers, who remained the same raters throughout the study to ensure consistency across assessments. The SDQ consists of 25 items, distributed across five subscales of five items each: emotional symptoms, behavioral problems, peer relationship problems, hyperactivity/inattention, and prosocial behavior. Each item is answered on a three-point scale: 0 (not true), 1 (somewhat true), and 2 (completely true). All SDQ subscales were used in this study. The subscales for emotional symptoms, behavioral problems, peer relationship problems, and hyperactivity/inattention have a score range of 0 to 10 points, with higher scores indicating poorer school adjustment. The prosocial behavior subscale also ranges from 0 to 10 points, with higher scores indicating better school adjustment. Scores for each subscale were used as continuous variables in the statistical analyses. The SDQ has demonstrated adequate psychometric properties in children and school-aged children and, in this study, showed adequate internal consistency, as assessed by Cronbach’s alpha coefficient (*α* = 0.912).

### Intervention

Participants assigned to the experimental group took part in a combined physical–cognitive intervention integrating soroban abacus training with structured physical exercise. The program was implemented during the 2024–2025 academic year within regular Physical Education lessons conducted in the school gymnasium. The intervention lasted 12 weeks and consisted of two sessions per week. A detailed session-by-session description of the intervention protocol, including examples of motor games and soroban exercises, is provided in Appendix A.

Each session lasted approximately 60 min and was structured into three phases: (i) warm-up phase (5 min); (ii) main phase (45 min); and (iii) review and feedback phase (10 min).

During the warm-up phase, participants performed dynamic exercises designed to progressively activate the musculoskeletal system and prepare the children for the cognitive–motor demands of the session. Activities included dynamic stretching movements, locomotor exercises, short hopping tasks, light running, coordination activities, and animal-movement imitation exercises aimed at stimulating balance, mobility, and general coordination. During this phase, children also reviewed the correct manipulation techniques for the soroban abacus, including the use of the thumb to raise beads, the index finger to lower beads, and the exclusive use of the index finger to manipulate the upper bead representing the value of five. Previously learned calculations were reviewed at the beginning of each session to reinforce procedural learning continuity.

The main phase integrated soroban-based cognitive training with cooperative motor games designed to stimulate executive functioning, coordination, balance, strength, and self-regulation. Cognitive and motor tasks were alternated within the same session rather than implemented as independent blocks, thereby promoting continuous interaction between physical and cognitive demands. Soroban instruction followed a progressive sequence adapted to the developmental level of the participants: (a) Sessions 1–3 focused on familiarization with the soroban structure, bead values, and single-digit addition and subtraction without carrying procedures; (b) Sessions 4–5 introduced mental visualization of the abacus and direct mental calculations; (c) Sessions 6–7 focused on “number friends” (complementary numerical combinations such as 1 + 4 = 5) to facilitate operations when insufficient beads were available; (d) Sessions 8–9 applied “number friends” to subtraction situations; (e) Sessions 10–11 progressed to two-digit addition and subtraction using multiple soroban rows; and (f) Sessions 12–14 focused on three-digit operations and progressively faster mental calculations.

Simultaneously, participants engaged in 2–3 motor games per session selected according to play-based and cooperative learning principles. Examples included relay races involving object transport, balance and obstacle circuits, rhythm and synchronization activities, reaction games using visual or auditory cues, and cooperative movement challenges requiring decision-making and inhibitory control. The complexity of the motor tasks progressively increased throughout the intervention in terms of coordination demands, cognitive engagement, and rule adaptation. Following each physical activity, children completed short soroban exercises designed to reinforce the integration of motor and cognitive processing.

The final phase of each session focused on review, feedback, and consolidation of learning. Children discussed task performance, reflected on difficulties encountered during the activities, and reviewed calculation strategies and motor challenges completed during the session. Group discussions encouraged emotional expression, self-reflection, and peer interaction. Attendance and participation were monitored throughout the intervention, and communication with classroom tutors and legal guardians was maintained to support adherence to the program and preserve intervention fidelity. S*ample size calculation.*

An *a priori* sample size calculation was performed using G*Power 3.1.9.7. The calculation was based on a repeated-measures ANOVA with a within–between interaction, considering two groups (experimental and control) and two measurement points (pre- and post-intervention). Assuming a small-to-moderate effect size (*f* = 0.16), an alpha level of 0.05, a statistical power of 0.80, a correlation among repeated measures of 0.50, and a nonsphericity correction of 1, the minimum required sample size was 80 participants. The final analyzed sample consisted of 82 participants, exceeding the minimum sample size required for the study.

### Procesure

To recruit participants, an educational institution was contacted and an initial meeting was organized with the students’ legal guardians, during which detailed information about the study was provided. Guardians received an information sheet and informed consent forms. Once the signed consent forms were obtained and the eligibility criteria were verified, baseline data collection began. The study was preregistered at ClinicalTrials.gov (Identifier: NCT06663540).

Participants were then randomly assigned to one of two groups: (i) Experimental Group (EG): this group participated in a combined program incorporating the use of the soroban abacus and physical exercises, conducted in the school gymnasium during Physical Education classes (from 12:00 to 12:45 p.m.); and (ii) Control Group (CG): this group followed the regular Physical Education curriculum without any additional intervention.

Randomization was performed in a 1:1 ratio using a computer-generated table of random numbers. Allocation concealment was ensured through the use of numbered, sealed, opaque envelopes prepared and managed by an independent researcher not involved in participant recruitment, intervention delivery, outcome assessment, or statistical analysis. The envelopes were securely stored and opened only after completion of the baseline assessments.

Blinding procedures were implemented whenever feasible within the educational setting. Participants, teachers, and outcome assessors were not informed of group allocation during the baseline data collection phase. However, due to the nature of the intervention, participants and instructors became aware of group allocation during the implementation of the program. The independent researcher responsible for administering the outcome measures and conducting the statistical analyses remained blinded to group assignment throughout the assessment process.

The intervention sessions were delivered by the students’ instructors, who received training in the program methodology during a 2-week preparatory phase before the intervention began. Throughout the study, members of the research team supervised the sessions to ensure that the planned activities were implemented correctly. To monitor engagement and adherence, attendance and observation records were maintained for each session. Statistical analyses followed a per-protocol approach. Participants who attended less than 80% of the scheduled sessions or who did not complete the post-intervention assessments were excluded from the final analyses, as insufficient attendance or incomplete data could compromise the reliability and validity of the results. Missing data were minimal and corresponded to two participants from the control group who did not complete the post-test assessment; therefore, no imputation procedures were applied.

### Data analysis

Continuous and categorical variables were analyzed according to group assignment (experimental group vs. control group) using Student’s t-test and chi-square test, respectively. Before conducting the analyses, normality and homogeneity assumptions were assessed using the Kolmogorov–Smirnov test and Levene’s test, respectively. To examine the effects of the intervention, separate mixed analyses of variance (ANOVA) were conducted for each outcome variable. Group assignment (experimental group vs. control group) was included as the between-subjects factor, and assessment time (pre-intervention vs. post-intervention) as the within-subjects factor. Outcome variables included self-regulation assessed with the Self-Regulation Questionnaire for Children (SRQ-C); emotional regulation and lability/negativity assessed with the Emotion Regulation Checklist (ERC); and school adjustment dimensions assessed with the Strengths and Difficulties Questionnaire (SDQ), including emotional symptoms, behavioral problems, peer relationship problems, hyperactivity/inattention, and prosocial behavior. Self-regulation assessed with the SRQ-C and emotional regulation assessed with the ERC were predefined as the primary outcomes of the study, as they were directly related to the main objectives of the intervention. In contrast, all SDQ dimensions were considered secondary exploratory outcomes and were analyzed for hypothesis-generating purposes only. Given the exploratory nature of these analyses and the relatively small sample size, formal correction for multiple comparisons was not applied, as overly conservative procedures may increase the risk of Type II errors in pilot studies. Therefore, SDQ-related findings should be interpreted cautiously and considered preliminary. The interaction between group and assessment time was also examined to determine whether changes over time differed between groups. Effect sizes for within-group comparisons were estimated using dz., whereas Hedges’ *g* was calculated for between-group comparisons. Values ≤ 0.20 were considered small, 0.50 moderate, and ≥ 0.80 large. Partial eta squared (*η*^2^) was also reported for ANOVA analyses. Statistical significance was established at *p* < 0.05. Data analyses were performed using SPSS software, version 17.0 (SPSS Inc., Chicago, IL, USA).

## Results

### Self-regulation

**Figure 2:**
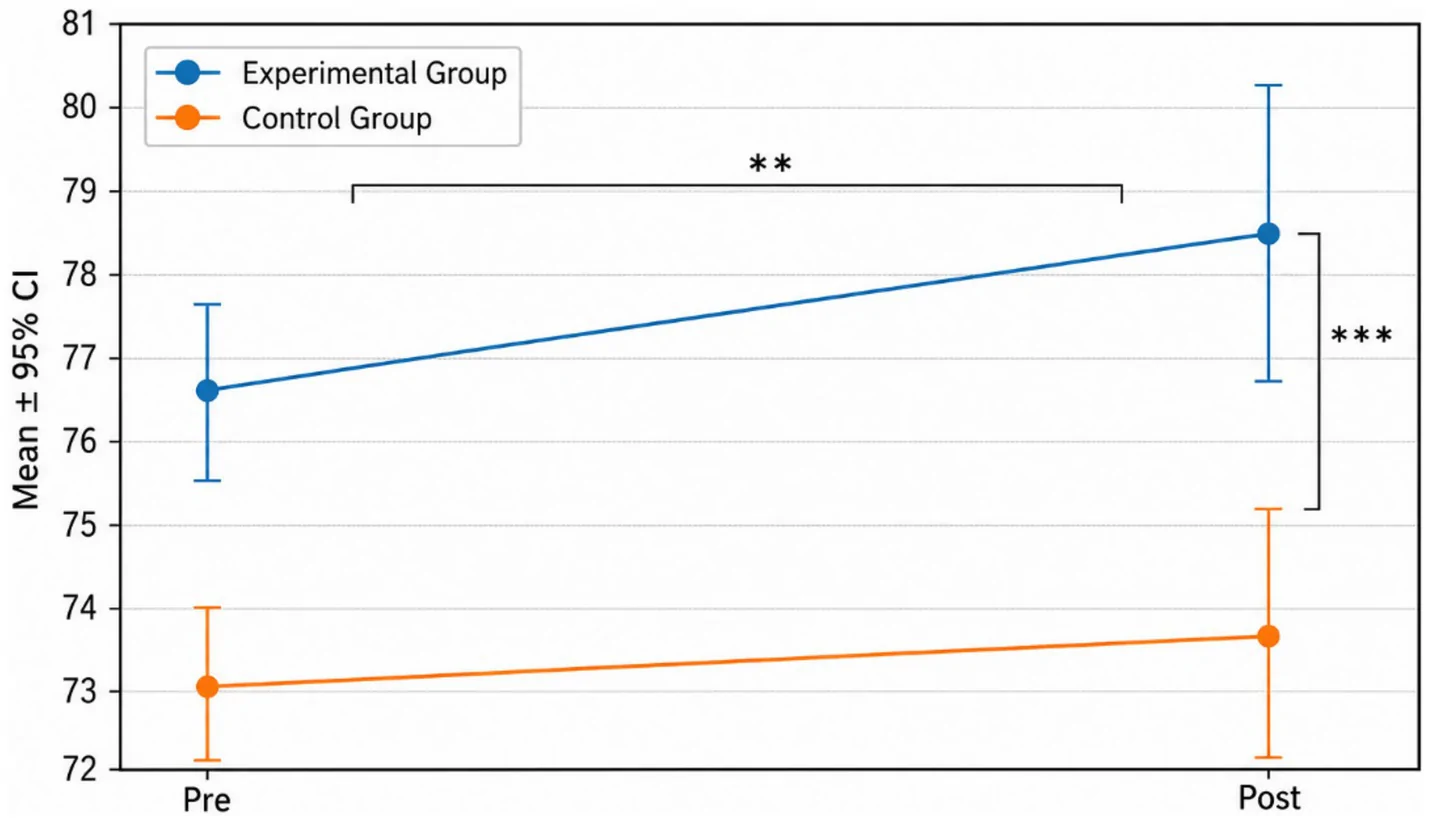
Self-regulation scores before and after the intervention. Values represent means ± 95% confidence intervals. The experimental group showed a significant increase in self-regulation from pre- to post-intervention (**p = 0.002). Post-intervention self-regulation scores were significantly higher in the experimental group than in the control group (***p < 0.001).

The experimental group showed a significant improvement in self-regulation from baseline to post-intervention, *t*(40) = −3.393, *p* = 0.002, dz. = 0.53, 95% CI [0.19, 0.86]. In addition, post-intervention self-regulation scores were significantly higher in the experimental group compared with the control group, *t*(80) = 4.187, *p* < 0.001, Hedges’ *g* = 0.91, 95% CI [0.45, 1.36]. These pre–post changes for both groups are illustrated in Figure 2.

### Emotional regulation

**Figure 3:**
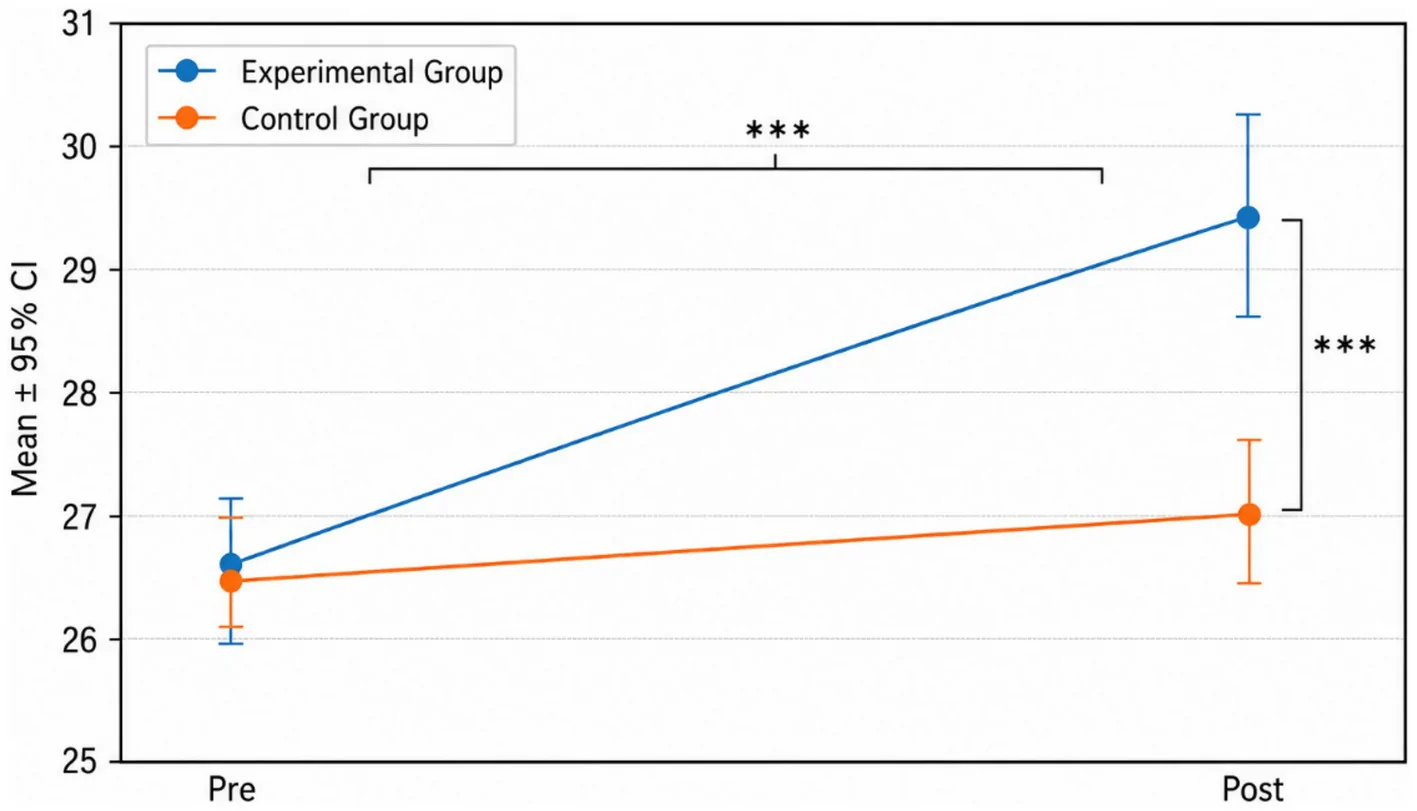
Pre- and post-intervention ERC emotional regulation scores in the experimental group and control group. Values represent means ± 95% confidence intervals. The experimental group showed a significant increase in emotional regulation from pre- to post-intervention (***p < 0.001). Post-intervention emotional regulation scores were significantly higher in the experimental group than in the control group (***p < 0.001).

Regarding emotional regulation, the experimental group showed significant improvements from baseline to post-intervention, *t*(40) = −9.390, *p* < 0.001, dz. = 1.47, 95% CI [0.98, 1.96]. In addition, post-intervention emotional regulation scores were significantly higher in the experimental group compared with the control group, *t*(80) = 5.752, *p* < 0.001, Hedges’ *g* = 1.26, 95% CI [0.79, 1.72]. These pre–post changes for both groups are illustrated in Figure 3.

**Figure 4:**
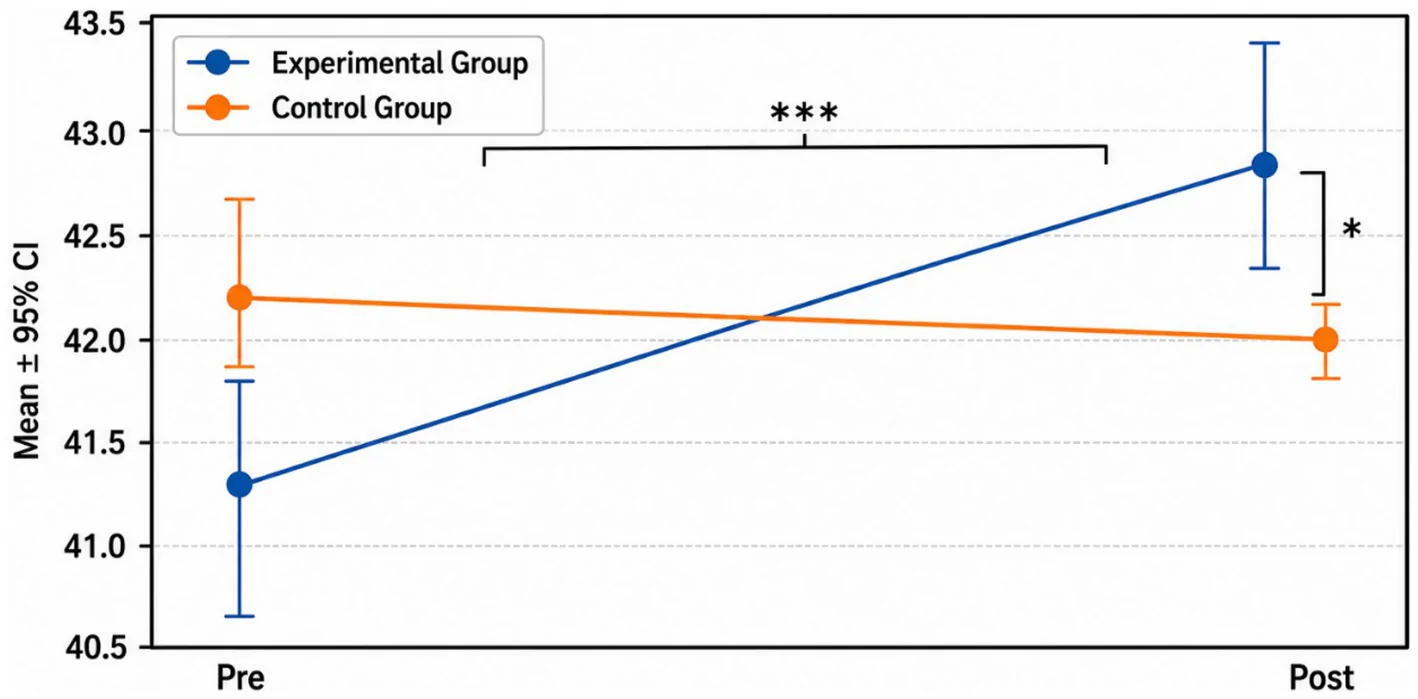
Pre- and post-intervention ERC lability/negativity scores in the experimental group and control group. Data are presented as means ± 95% confidence intervals. The experimental group showed a significant increase in lability/negativity from pre- to post-intervention (***p < 0.001). Post-intervention lability/negativity scores were significantly higher in the experimental group than in the control group (*p = 0.010).

For lability/negativity, the experimental group also showed significant changes from baseline to post-intervention, *t*(40) = −3.989, *p* < 0.001, dz. = 0.62, 95% CI [0.27, 0.97]. Furthermore, significant between-group differences were observed at post-intervention, *t*(80) = 2.635, *p* = 0.010, Hedges’ *g* = 0.58, 95% CI [0.14, 1.01]. These pre–post changes for both groups are illustrated in Figure 4.

### School adjustment

**Figure 5:**
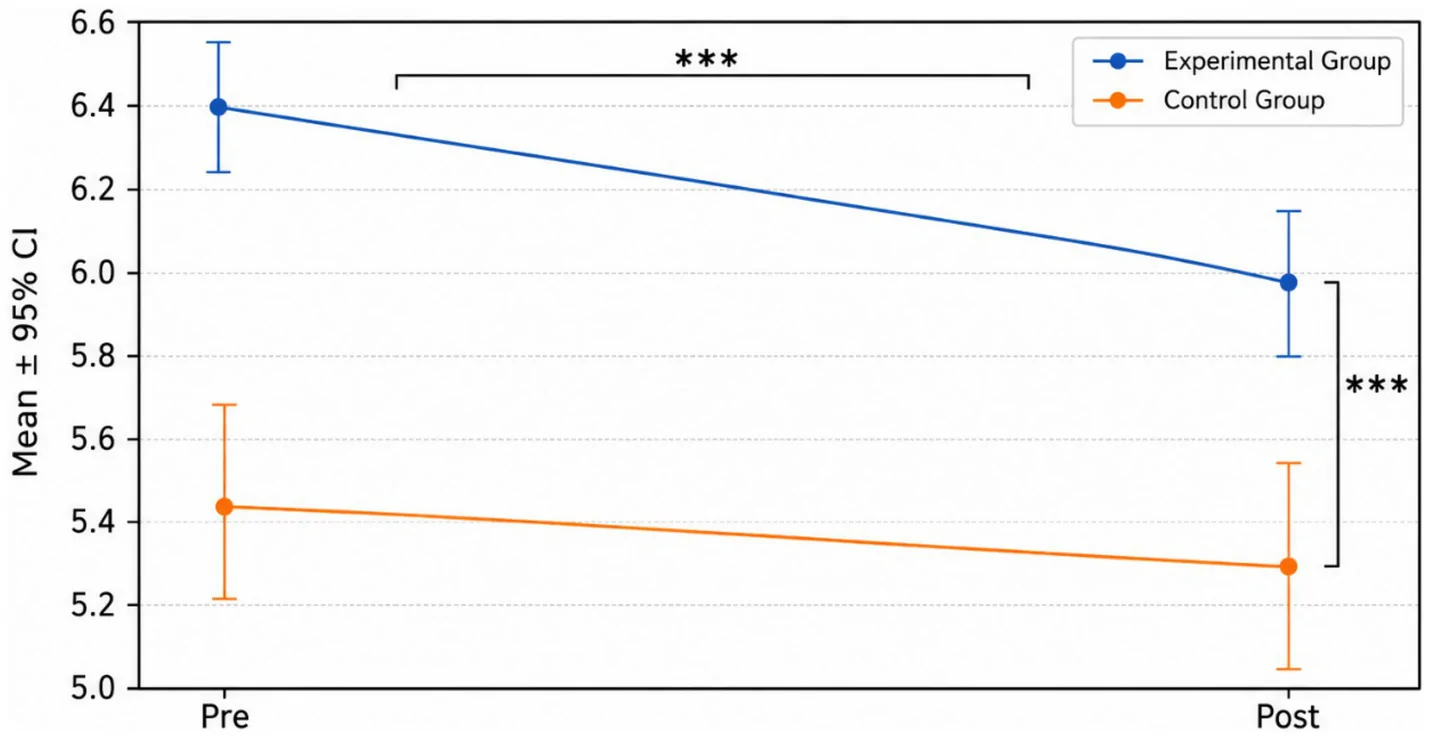
Pre- and post-intervention SDQ emotional symptoms scores in the experimental group and control group. Data are presented as means ± 95% confidence intervals. The experimental group showed a significant increase in emotional symptoms from pre- to post-intervention (***p < 0.001). Post-intervention emotional symptoms scores were significantly higher in the experimental group than in the control group (***p < 0.001).

Emotional symptoms showed significant improvements in the experimental group from baseline to post-intervention, *t*(40) = 4.794, *p* < 0.001, dz. = 0.75, 95% CI [0.39, 1.11]. In addition, post-intervention emotional symptoms scores differed significantly between groups, *t*(80) = 3.814, *p* < 0.001, Hedges’ *g* = 0.83, 95% CI [0.38, 1.28]. These pre–post changes for both groups are illustrated in Figure 5.

**Figure 6:**
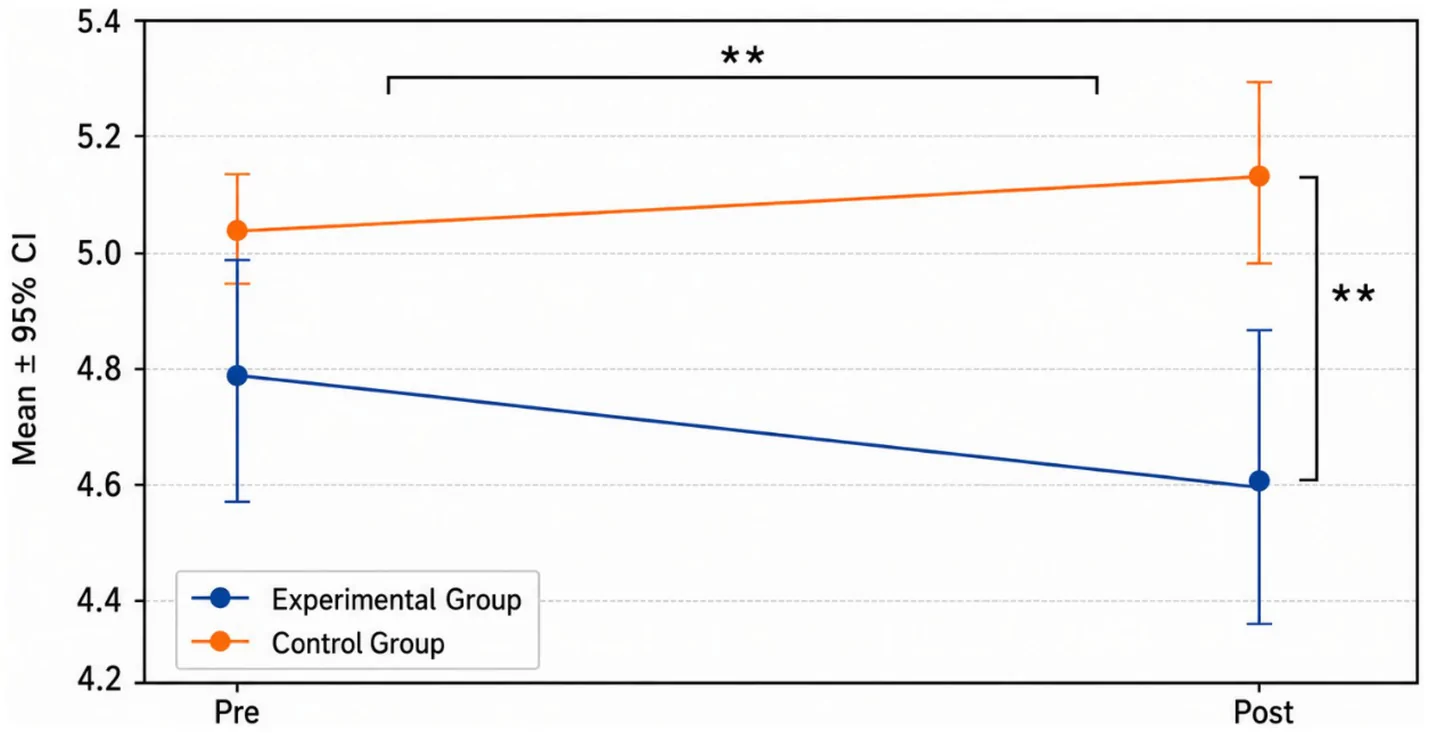
Pre- and post-intervention SDQ behavioral problems scores in the experimental group and control group. Data are presented as means ± 95% confidence intervals. The experimental group showed a significant increase in behavioral problems scores from pre- to post-intervention (**p < 0.01). Post-intervention behavioral problems scores were significantly higher in the experimental group than in the control group (**p < 0.01).

Behavioral problems also showed significant changes in the experimental group following the intervention, *t*(40) = 3.327, *p* = 0.002, dz. = 0.52, 95% CI [0.18, 0.86]. Significant between-group differences were observed at post-intervention, *t*(80) = 3.424, *p* = 0.001, Hedges’ *g* = 0.75, 95% CI [0.30, 1.19]. These pre–post changes for both groups are illustrated in Figure 6.

**Figure 7:**
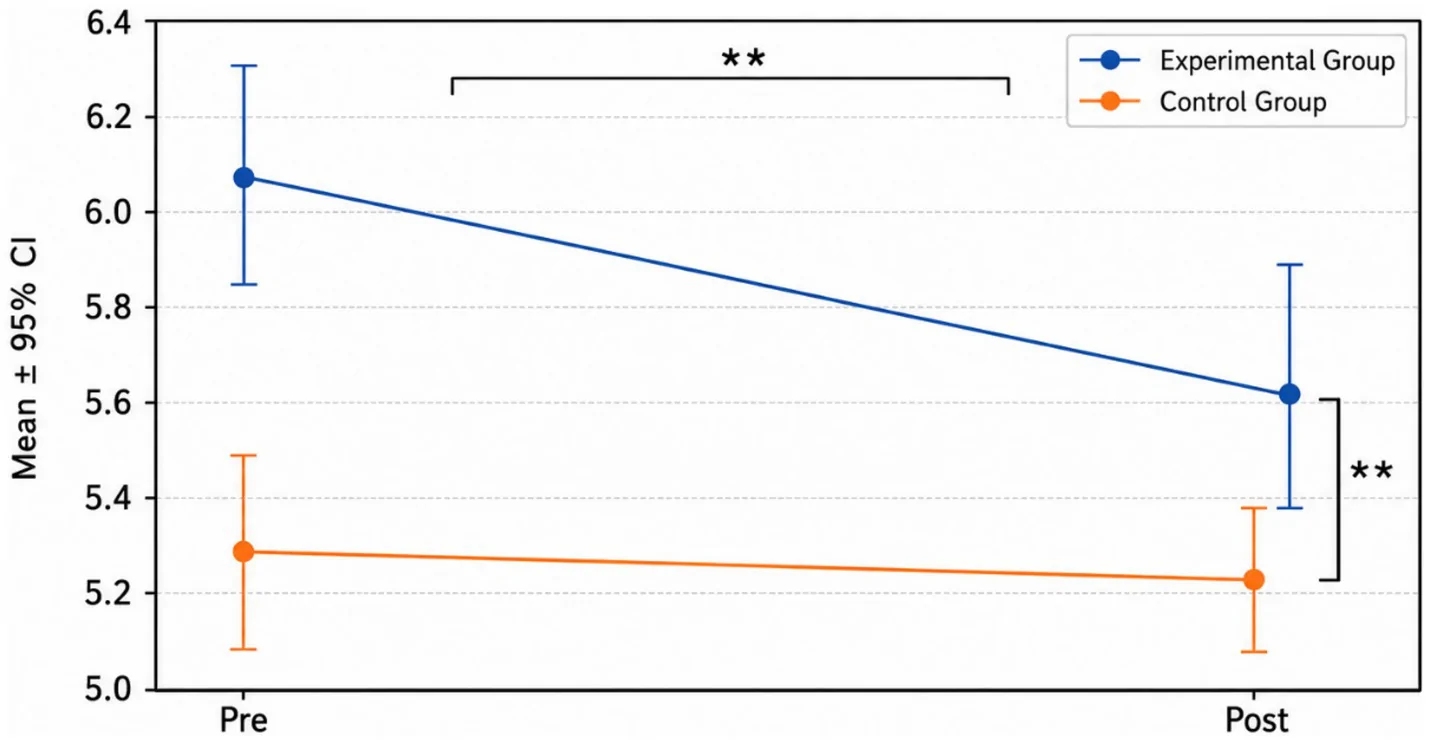
Pre- and post-intervention SDQ peer relationship problems scores in the experimental group and control group. Data are presented as means ± 95% confidence intervals. The experimental group showed a significant increase in peer relationship problems scores from pre- to post-intervention (**p < 0.01). Post-intervention peer relationship problems scores were significantly higher in the experimental group than in the control group (**p < 0.01).

Similarly, peer relationship problems showed significant improvements from pre- to post-intervention in the experimental group, *t*(40) = 3.036, *p* = 0.004, dz. = 0.47, 95% CI [0.13, 0.81]. Significant differences between the experimental and control groups were also observed at post-intervention, *t*(80) = 2.689, *p* = 0.009, Hedges’ *g* = 0.59, 95% CI [0.15, 1.02]. These pre–post changes for both groups are illustrated in Figure 7.

**Figure 8:**
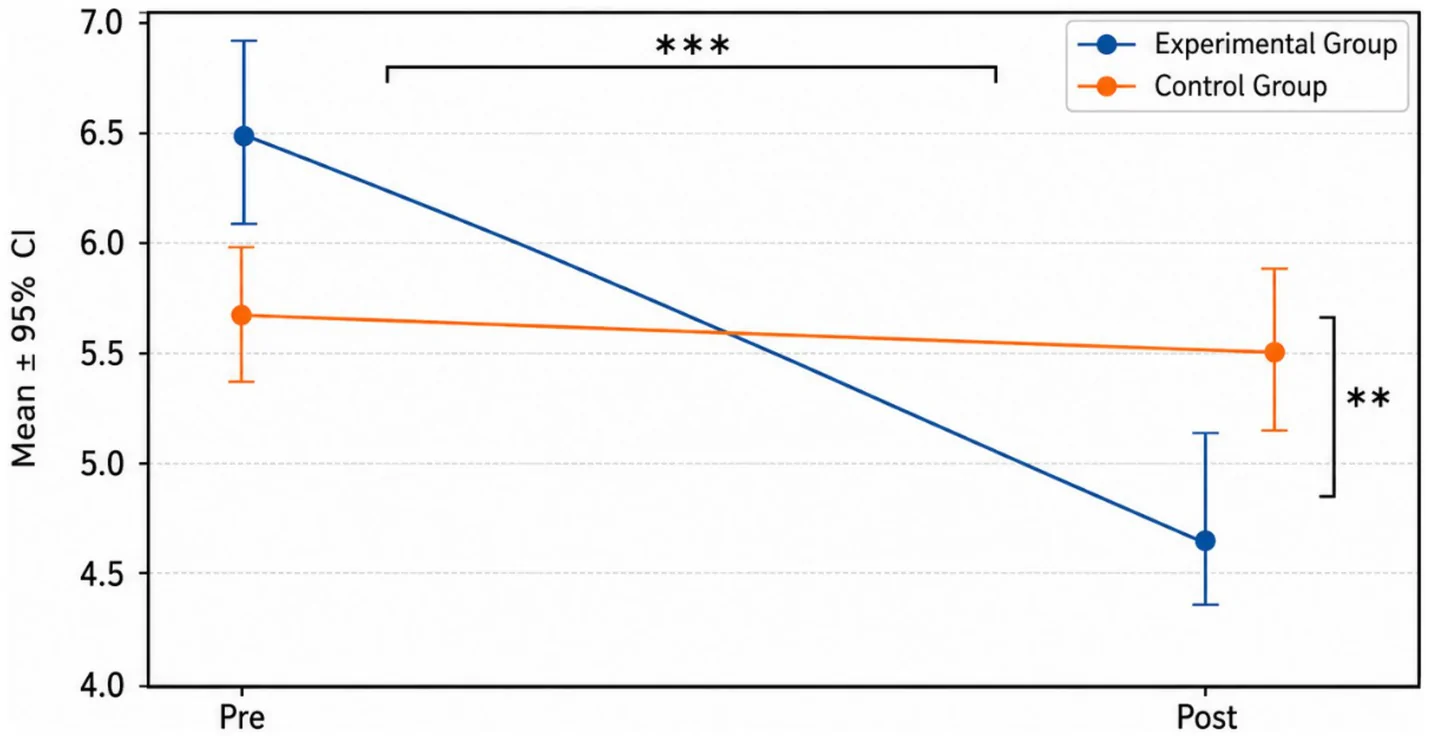
Pre- and post-intervention SDQ hyperactivity/inattention scores in the experimental group and control group. Data are presented as means ± 95% confidence intervals. The experimental group showed a significant increase in hyperactivity/inattention scores from pre- to post-intervention (***p < 0.001). Post-intervention hyperactivity/inattention scores were significantly higher in the experimental group than in the control group (**p = 0.003).

Hyperactivity/inattention also improved significantly in the experimental group from baseline to post-intervention, *t*(40) = 7.904, *p* < 0.001, dz. = 1.23, 95% CI [0.77, 1.69]. Significant post-intervention differences were additionally found between groups, *t*(80) = 3.064, *p* = 0.003, Hedges’ *g* = 0.67, 95% CI [0.23, 1.11]. These pre–post changes for both groups are illustrated in Figure 8.

**Figure 9:**
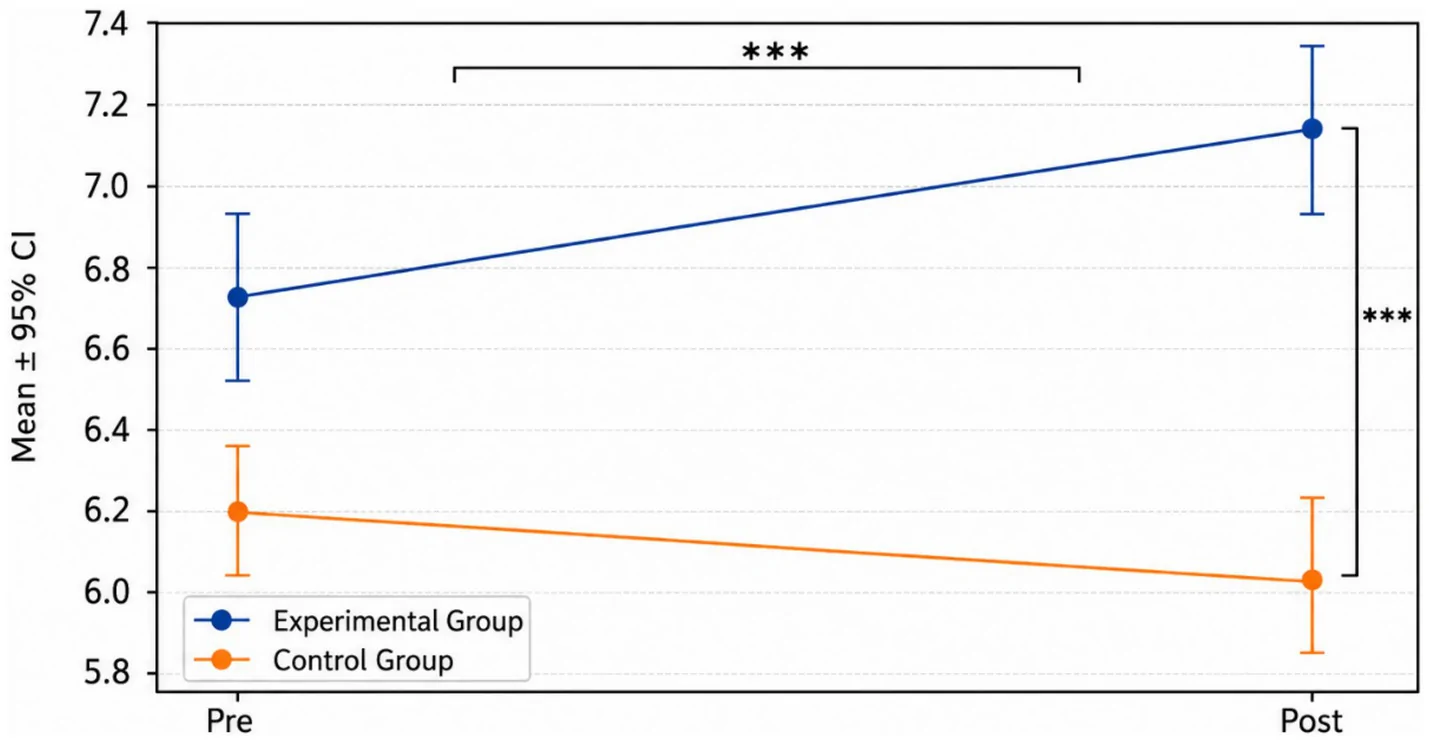
Pre- and post-intervention SDQ prosocial behavior scores in the experimental group and control group. Data are presented as means ± 95% confidence intervals. The experimental group showed a significant increase in hyperactivity/inattention scores from pre- to post-intervention (***p < 0.001). Post-intervention hyperactivity/inattention scores were significantly higher in the experimental group than in the control group (***p < 0.001).

Finally, prosocial behavior improved significantly in the experimental group following the intervention, *t*(40) = 4.141, *p* < 0.001, dz. = 0.65, 95% CI [0.30, 1.00]. Likewise, the between-group comparison at post-intervention revealed significant differences favoring the experimental group, *t*(80) = 7.634, *p* < 0.001, Hedges’ *g* = 1.67, 95% CI [1.16, 2.18]. These pre–post changes for both groups are illustrated in Figure 9.

In addition, Table 2 summarizes a broader and more consistent pattern of the intervention’s effects on all assessed outcomes, reflecting both changes over time and differences between groups and their interactions.

**Table 2:** Effects of the intervention on emotional regulation and school adjustment.

Variables	EG (*n* = 41)		CG (*n* = 41)		Group			Time			Group × Time		
	Pre	Post	Pre	Post	*F*(79)	*p*-value	*η* ^2^	*F*(79)	*p*-value	*η* ^2^	*F*(79)	*p*-value	*η* ^2^
Self-regulation	75.56 ± 3.79	78.46 ± 5.69	73.04 ± 2.80	73.66 ± 4.64	20.738	*p* < 0.001	0.206	11.064	0.001	0.121	4.603	0.035	0.054
ERC_Emotional regulation	26.57 ± 1.94	29.45 ± 2.23	26.47 ± 1.68	26.99 ± 1.60	11.698	0.001	0.128	89.174	*p* < 0.001	0.527	43.472	*p* < 0.001	0.352
ERC_Lability/negativity	41.35 ± 2.37	42.84 ± 1.80	42.23 ± 1.47	42.05 ± 0.66	0.022	0.882	0.000	9.083	0.003	0.102	14.443	*p* < 0.001	0.153
SDQ_ Emotional symptoms	6.41 ± 0.54	5.95 ± 0.70	5.43 ± 0.76	5.29 ± 0.85	35.997	*p* < 0.001	0.310	13.650	*p* < 0.001	0.146	4.217	0.043	0.050
SDQ_ Behavioral problems	4.79 ± 0.71	4.62 ± 0.81	5.04 ± 0.15	5.12 ± 0.45	9.311	0.003	0.104	1.178	0.281	0.015	8.477	0.005	0.096
SDQ_ Peer relationship problems	6.07 ± 0.61	5.64 ± 0.84	5.28 ± 0.71	5.25 ± 0.37	28.766	*p* < 0.001	0.264	5.847	0.018	0.068	4.341	0.040	0.051
SDQ_ Hyperactivity/inattention	6.47 ± 0.97	4.79 ± 1.22	5.68 ± 0.99	5.56 ± 1.03	0.007	0.934	0.000	46.539	*p* < 0.001	0.368	34.767	*p* < 0.001	0.303
SDQ_ Prosocial behavior	6.71 ± 0.63	7.13 ± 0.71	6.20 ± 0.47	6.04 ± 0.56	48.628	*p* < 0.001	0.378	3.882	0.052	0.046	18.154	*p* < 0.001	0.185

Quantitative variables are presented as mean and standard deviation. EG, experimental group; CG, control group; ERC, Emotion Regulation Checklist; SDQ, the Strengths and Difficulties Questionnaire.

## Discussion

The aim of this study was to analyze the impact of an intervention based on motor games and the soroban abacus on self-regulation, emotional regulation, and school adjustment in children in the first cycle of primary education. Overall, the results provide preliminary empirical support for the potential effectiveness of this integrative approach, showing significant improvements in all variables assessed in the experimental group compared to the control group. These findings suggest the potencial relevance of multimodal interventions in early educational settings from a neuroeducational perspective.

One of the main findings of the study is the significant improvement in self-regulation among the children who participated in the combined program. The results show moderate to large effects in both within-group and between-group analyses, suggesting that the intervention was effective in enhancing skills related to planning, behavioral control, task persistence, and self-monitoring. From the perspective of cognitive neuroscience, self-regulation relies on the functioning of executive functions, especially working memory, inhibitory control, and cognitive flexibility—processes closely linked to the development of the prefrontal cortex during childhood (Segundo-Marcos et al., 2022). In this sense, the combination of structured physical activities with complex cognitive tasks, such as using the soroban, may have generated simultaneous stimulation of these systems, promoting functional training of self-regulation (Rondão et al., 2022). Previous studies have indicated that physical activities involving high cognitive demands produce greater executive benefits than unstructured physical activity, as they require decision-making, attentional control, and constant adaptation to environmental rules (Mandolesi et al., 2018; Du et al., 2025). Furthermore, the progressive use of the abacus demands a high degree of sustained attentional control, sequential planning, and error self-correction, which contributes to the development of cognitive self-regulation. These results are consistent with research demonstrating that cognitive training with the abacus not only improves mathematical performance but also metacognitive and self-regulatory processes in children (Na et al., 2015; Mou et al., 2022).

Regarding emotional regulation, the results indicate significant improvements in the ERC’s emotional regulation subscale and a reduction in lability/negativity in the experimental group, with large effect sizes. These data suggest that the intervention not only fostered a greater capacity to modulate and express emotions adaptively, but also contributed to decreasing emotional instability and intense negative responses. From a socio-emotional development perspective, emotional regulation is a key component for school adjustment and psychological wellbeing (Martínez-Líbano et al., 2025). Participation in cooperative motor games offers natural opportunities to experience intense emotions (frustration, enthusiasm, competition), learn to manage and regulate them in a structured social environment (Marker and Staiano, 2015). At the same time, the cognitive component of the program demands frustration tolerance, impulse control, and persistence in the face of difficulty—skills directly related to emotional regulation. These results align with recent research highlighting the effectiveness of integrated interventions in improving emotional regulation and reducing maladaptive behaviors in childhood (Moltrecht et al., 2021; Saccaro et al., 2024). Furthermore, training in contexts that combine cognitive and physical demands can foster greater emotional awareness and a better appraisal of emotional situations—key aspects identified by Lopes et al. (2011) as predictors of higher-quality social interactions. Therefore, these findings are particularly relevant, as they suggest that educational programs integrating physical and cognitive activation can positively influence the neural systems involved in emotional regulation, contributing to more balanced emotional development in early childhood.

Regarding school adjustment, the experimental group showed significant improvements in all dimensions assessed using the SDQ: reduction in emotional symptoms, behavioral problems, difficulties in peer relationships, and hyperactivity/inattention, as well as an increase in prosocial behavior. The observed effect sizes, especially for hyperactivity/inattention and prosocial behavior, suggest potentially meaningful practical effects; however, these findings should be interpreted cautiously given the sample size and exploratory nature of some analyses. These results support the literature linking self-regulation and emotional regulation with better school adjustment and a more positive adaptation to the educational context (Muñoz-Troncoso and Riquelme-Mella, 2025; Da Costa Dutra et al., 2023). The decrease in hyperactivity and inattention can be interpreted as a direct consequence of the strengthening of inhibitory control and sustained attention (Bozhilova et al., 2018), skills systematically trained during the program. Similarly, the increase in prosocial behavior can be attributed to the cooperative nature of the motor games and group dynamics, which foster empathy, collaboration, and respect for rules (Ouyang et al., 2025). Previous studies have demonstrated that social–emotional learning programs and interventions based on structured physical activity have positive effects on prosocial behavior and school coexistence (Tang et al., 2025; Zhang et al., 2024). In this sense, the results of the present study expand upon this evidence by showing that the explicit integration of a complex cognitive component enhances these effects, contributing to an overall improvement in school adjustment. However, given the exploratory nature of the SDQ analyses and the absence of formal correction for multiple comparisons, these findings should be interpreted with caution.

An important contribution of the present study lies in the integrative nature of the intervention. While previous physical–cognitive programs have generally examined executive functioning, attention, or academic performance, the current intervention combined cooperative motor games with progressive soroban-based cognitive training specifically aimed at promoting self-regulation, emotional regulation, and school adjustment within the same educational framework. This multidimensional approach may explain the broad pattern of improvements observed across cognitive, emotional, and behavioral domains, supporting the relevance of integrated neuroeducational interventions during early primary education. From an applied perspective, the findings of this study have important implications for the design of educational programs in primary education. The proposed intervention is feasible in real school settings, easily integrated into the physical education curriculum, and promotes learning transferable to other academic and social areas. Furthermore, it responds to current demands in neuroeducation, which advocates for pedagogical approaches based on scientific evidence regarding brain function and child development.

Despite the robustness of the results, the study has some limitations that should be considered. First, the sample size, while adequate for detecting significant effects, limits the generalizability of the findings. Second, the absence of follow-up assessments prevents determining the long-term stability and maintenance of the observed effects over time. Future longitudinal studies should incorporate delayed post-intervention measurements to evaluate the persistence of improvements in self-regulation, emotional regulation, and school adjustment. Furthermore, the use of a per-protocol analytical approach rather than an intention-to-treat analysis may have introduced attrition-related bias and could limit the generalizability of the findings. Future studies should consider implementing intention-to-treat analyses to provide more robust estimates of intervention effectiveness. Furthermore, the use of questionnaires completed by adults, although validated, may be subject to perception and evaluation bias. In addition, multiple secondary analyses were conducted across the different SDQ dimensions without formal multiplicity correction. Although formal correction procedures for multiple comparisons were not applied due to the exploratory nature of the secondary outcomes and the limited sample size, this decision may increase the probability of Type I error. Therefore, the SDQ-related findings should be interpreted cautiously until replicated in larger confirmatory studies.

## Conclusion

In conclusion, the results of this study suggest that a physical–cognitive intervention based on motor games and soroban abacus training may be effective in improving self-regulation, emotional regulation, and school adjustment in primary school children. These findings provide preliminary evidence for the field of Mind, Brain, and Education by supporting the potential value of integrated educational approaches that combine cognitive and motor stimulation within school settings. The results also highlight the potential usefulness of structured physical–cognitive programs for promoting socio-emotional development and adaptive functioning during early primary education. However, given the relatively small sample size and the exploratory nature of some analyses, the findings should be interpreted cautiously until replicated in larger confirmatory studies.

## Data Availability

The raw data supporting the conclusions of this article will be made available by the authors, without undue reservation.
