# Supplementary material for: Impact of a physical–cognitive intervention on emotional regulation and school adjustment in primary school children
Source: Front Psychol. 2026 Aug 13;17:1790797. doi: 10.3389/fpsyg.2026.1790797 (PMC13518122; doi:10.3389/fpsyg.2026.1790797)
Supplement: Supplementary file 1 [file Table_1.DOCX]

**Appendix A. Detailed Physical–Cognitive Intervention Protocol**

**Overview of the Intervention**

The intervention consisted of a 12-week physical–cognitive program implemented during regular Physical Education classes. Sessions were conducted twice per week and lasted approximately 60 minutes. The intervention combined structured motor games with progressive cognitive training using the soroban abacus.

Each session followed the same general structure:

1. **Warm-up phase (5 minutes)**
   - Dynamic mobility exercises.
   - Coordination activation tasks.
   - Review of soroban finger placement and manipulation techniques.
2. **Main phase (45 minutes)**
   - Combination of motor games and soroban-based cognitive exercises.
   - Progressive increase in motor coordination and cognitive load.
   - Cooperative and play-based learning approach.
3. **Cool-down and correction phase (10 minutes)**
   - Review of mathematical exercises.
   - Group discussion and feedback.
   - Reflection on task performance and emotional regulation.

**Session-by-Session Protocol**

| Sessions | Soroban Cognitive Training | Cognitive Load | Motor Games | Motor Objectives |
| --- | --- | --- | --- | --- |
| 1–2 | Introduction to the soroban: identification of beads, columns, and numerical values. Single-digit addition without carrying. | Low | Relay races with object transport; balance circuits. | Basic coordination, dynamic balance, spatial orientation. |
| 3–4 | Single-digit subtraction without borrowing. Finger movement automation. | Low–moderate | Cooperative ball passing games; reaction games with color cues. | Visuomotor coordination, reaction speed, cooperation. |
| 5–6 | Mental visualization of the abacus for direct addition. | Moderate | Obstacle circuits with memory sequences. | Working memory, coordination, inhibitory control. |
| 7–8 | Introduction to “number friends” (complementary numbers summing to 5 or 10). | Moderate | Team-based strategy games requiring movement decisions. | Cognitive flexibility, decision making, teamwork. |
| 9–10 | Application of “number friends” in subtraction tasks. | Moderate–high | Rhythm and synchronization games; mirror movement tasks. | Attention, synchronization, emotional control. |
| 11–12 | Two-digit addition and subtraction using multiple soroban rows. | High | Multi-station cooperative circuits with task switching. | Sustained attention, task switching, motor planning. |
| 13–14 | Three-digit operations and increased speed in mental calculations. | High | Competitive–cooperative team challenges with rule adaptation. | Executive control, adaptability, behavioral regulation. |
| 15–16 | Mixed operations combining addition and subtraction under time constraints. | High | Dynamic games involving rapid decision making and movement inhibition. | Inhibitory control, processing speed, self-regulation. |
| 17–18 | Mental calculation without physical abacus support. | High | Sequential memory movement games. | Working memory, concentration, coordination. |
| 19–20 | Complex mixed calculations with progressive reduction in response time. | High | Team problem-solving movement challenges. | Cooperative regulation, emotional management. |
| 21–22 | Consolidation of mental abacus operations and autonomous problem solving. | High | Integrated cooperative game circuits. | Self-regulation, persistence, emotional adaptation. |
| 23–24 | Final review and integrated cognitive–motor tasks. | High | Cooperative challenges combining all previous motor skills. | Global integration of cognitive and motor abilities. |

**Motor Games**

**Coordination Games**

**Crazy Traffic Light**

Children were instructed to start, stop, or change direction according to the color announced by the instructor. This activity was designed to improve visuomotor coordination, inhibitory control, and reaction speed.

**Obstacle Trails**

Participants completed sequences of movements involving cones, hoops, jumps, crawling tasks, and directional changes. The activity aimed to stimulate agility, body control, and spatial orientation.

**Number Relay Race**

Children alternated running toward a board where they had to place the correct number to complete a basic arithmetic operation before returning to their team.

**Targeted skills:**

- Visuomotor coordination
- Reaction speed
- Working memory
- Cooperative learning

**Balance Games**

**Acrobat Path**

Participants walked slowly along lines or ropes placed on the floor while maintaining postural stability and balance.

**Mathematical Statues**

When hearing a specific number, children froze in a particular posture associated with the result of a previous arithmetic operation, promoting concentration and postural control.

**Numerical Jump**

Based on the result of an abacus operation, participants jumped sequentially between numbered floor markers.

**Targeted skills:**

- Dynamic balance
- Concentration
- Postural control
- Spatial orientation

**Strength Games**

**Cooperative Pull**

Children worked in teams using elastic bands or ropes to generate resistance while coordinating movements cooperatively.

**Push and Solve**

In pairs, participants gently pushed against each other’s palms while simultaneously solving simple mental arithmetic problems.

**Mathematical Carry**

Children transported lightweight sandbags to a designated location where they verbally solved an arithmetic problem before returning.

**Targeted skills:**

- Upper-body strength
- Cooperative effort
- Cognitive–motor integration
- Self-regulation

These motor games were implemented within a playful and cooperative learning environment specifically designed to integrate physical activity with soroban-based cognitive training. After completing each motor activity, participants solved mathematical problems using the abacus in order to reinforce the interaction between movement, executive functioning, and mathematical learning.

**Examples of Soroban Exercises**

**Basic Addition**

Example:

- 3 + 2
- 4 + 1

Children manipulated beads physically before progressing to mental visualization.

**Number Friends**

Example:

- 4 + 1 = 5
- 7 + 3 = 10

Children learned complementary numerical relationships to facilitate mental calculation.

**Two-Digit Operations**

Example:

- 24 + 15
- 38 − 12

Tasks required simultaneous use of multiple soroban rows.

**Mental Abacus Visualization**

Children solved operations mentally without physically manipulating the abacus.

Example:

- 46 + 28
- 72 − 35

These tasks progressively increased executive demands involving:

- Working memory
- Sustained attention
- Inhibitory control
- Cognitive flexibility

**Cognitive Load Progression**

The intervention followed a progressive increase in cognitive complexity:

1. Physical manipulation of beads.
2. Guided visual representation.
3. Autonomous mental calculation.
4. Time-constrained calculations.
5. Simultaneous cognitive–motor task integration.

This progression was designed to progressively stimulate executive functions and self-regulatory processes while maintaining age-appropriate task difficulty.
